# Characterization of the extra copy of TPOX locus with tri-allelic pattern

**DOI:** 10.1186/s12863-019-0723-2

**Published:** 2019-02-14

**Authors:** Qinrui Yang, Baonian Liu, Chengchen Shao, Yuxiang Zhou, Yining Yao, Yuyin Pan, Kuan Sun, Hongmei Xu, Chengtao Li, Ting Wei, Yueqin Zhou, Qiqun Tang, Jianhui Xie

**Affiliations:** 1 Department of Forensic Medicine, Shanghai Medical College of Fudan University, Shanghai, 200032 China; 2 Shanghai Key Laboratory of Forensic Medicine, Institute of Forensic Science, Ministry of Justice, Shanghai, 200063 China; 3 Novogene Co., LTD, Beijing, 100142 People’s Republic of China; 4 Department of Biochemistry and Molecular Biology, Shanghai Medical College of Fudan University, Shanghai, 200032 China

**Keywords:** Short tandem repeats, TPOX, Tri-allelic pattern, Recombination, Chromosome 2

## Abstract

**Background:**

An STR locus with tri-allelic pattern is occasionally observed in routine forensic casework. The extra copy of TPOX locus with tri-allelic pattern in populations has been assumed to be inserted into an X chromosome, which took place forth before the Bantu expansion in Africa. Nonetheless, the exact location of the duplication and the form of rearrangement in the human genome has not been clarified yet.

**Results:**

In this study, we investigated the extra copy of type 2 tri-allelic pattern at TPOX in various populations. While allele 10 is the major third allele in Africa, allele 11 appears more frequent in America and overwhelming in Chinese and Korean populations, which might attribute to the population substructures. Results from the investigation of family cases showed that the transmission of the extra allele had a similar genetic pattern of autosomal genes. Furthermore, a whole-genome sequencing followed by bioinformatics analysis revealed that the intact form of chromosomal duplication and rearrangement occurred ~ 407 kb away from the authentic TPOX locus on chromosome 2 in two cases. The breakpoints of the insertion were further validated in most other tri-allelic subjects, which can imply the identical origin from the ancestral extra copy. Nevertheless, de novo chromosomal duplication and rearrangement at thyroid peroxidase gene occur in populations.

**Conclusions:**

Instead of the extra allele 10 in African populations, the main third allele at TPOX with tri-allelic pattern is allele 11 in Chinese and Korean populations. The insertion of the extra copy into chromosome 2 occurs in most subjects with tri-allelic pattern at TPOX and demonstrates the transmission of the third allele from parents to offspring. The breakpoints of the ancestral extra copy are defined, which shows evidence of its inheritance from African populations. In addition, the simple validation method would help improve tri-allelic pattern calling, distinguish de novo chromosomal rearrangements, and also count the frequencies among different geographic regions. Therefore, the statistical interpretation of tri-allelic pattern at TPOX could be enhanced during forensic practice.

**Electronic supplementary material:**

The online version of this article (10.1186/s12863-019-0723-2) contains supplementary material, which is available to authorized users.

## Background

During STR typing for individual identification and paternity testing, the tri-allelic pattern, also named as three-banded pattern, is occasionally observed at a single STR locus on autosomal chromosomes. Up till now, a total of 337 cases of autosomal tri-allelic STRs have been reported at the STRBase website [1]. According to previous reports, tri-allelic patterns can be distinguished into type 1 and 2 patterns [2]. In type 1 pattern, the third allele presents different peak height from the other two alleles in STR typing and is concerned a result from slippage mutation in an early somatic cell development. In type 2 pattern, three alleles are of the same intensity with its generality going towards the duplication and chromosomal rearrangement. In fact, such duplication and chromosomal rearrangement can occur in both ancient and current populations. Therefore, tri-allelic patterns at different STR loci appear to have different configurations in populations.

Tri-allelic pattern at TPOX locus has been widely reported [3–17]. The frequency of TPOX tri-allelic pattern varies among populations, yet the parallel characteristics has been concluded. Lane et al. [5] revealed that the extra allele was almost always allele 10 in African populations and might be brought forth before the Bantu expansion. According to previous investigations in Dominican and Brazilian populations, tri-allelic cases at TPOX have been much frequently observed in females than in males. Besides, fathers with tri-allelic pattern at TPOX transmitted the third allele barely to their sons yet always to their daughters. Therefore, the extra copy was assumed to be inserted into an X chromosome. Nonetheless, it still remains unclear where exactly the extra copy lies in human genome.

To clarify the extra copy, we collected information of cases of type 2 tri-allelic pattern at TPOX from previous literature and samples from routine paternity testing in this study. The extra allele of TPOX together with its transmission from parents to offspring in Chinese populations was investigated. Furthermore, the location of the extra copy of TPOX in human genome was determined by whole genome sequencing together with bioinformatics analysis. An intact duplication and chromosomal rearrangement were revealed by detecting the breakpoint junctions.

## Methods

### Samples collection and STR genotyping

Samples were all collected from routine paternity tests among Chinese population. Genomic DNA was extracted by using Chelex-100 and proteinase K [18]. The polymerase chain reaction (PCR) was performed using PP21 (Promega, USA) and EX22 (AGCU, China) in a GeneAmp PCR System 9700 (Applied Biosystems, USA) according to the manufacturers’ recommendation. PCR products were separated by capillary electrophoresis in ABI PRISM 3130xL Genetic Analyzer (Applied Biosystems, USA). Allele designation was determined according to allelic ladders by using the GeneMapper® ID software v3.2 (Applied Biosystems, USA). A combined paternity index (CPI) was calculated within each family, where a CPI of at least 10,000 intended a true biological relationship. All procedures were approved by the ethics committee of Shanghai Medical College, Fudan University, and all individuals volunteered for this study based upon written informed consent.

### Collection of reported type 2 tri-allelic pattern at TPOX locus

Unrelated individual cases with tri-allelic patterns at TPOX locus were collected from STRBase categorized by geographic region. The extra allele was determined to be either allele 10 or allele 11. Under circumstances that both allele 10 and 11 occurred in the genotype calling of an individual, the case was excluded in this study. Following, searching of TPOX tri-allelic patterns both in individuals and among families was carried out in PubMed database and Chinese CNKI database using key words without any limitation including ‘TPOX’, ‘STR’, ‘three-banded pattern’, ‘tri-allelic pattern’, ‘tri-allelic variant’, ‘extra allele’, and/or ‘third allele’. Detailed information of TPOX tri-allelic patterns with allele combination, gender and population was collected.

### Whole genome sequencing, breakpoint analysis, and fusion gene prediction

A ~ 38× whole-genome sequencing (WGS) was performed using genomic DNA from two unrelated individuals with tri-allelic pattern at TPOX locus, respectively, on an Illumina X platform for paired read 150 bp (Novogene Bioinformatics Technology Co. Ltd., China). Valid raw read files were mapped to a reference sequence of human genome (hg19 build 37.3) as paired reads using Burrows-Wheeler Aligner (BWA) [19]. SAMtools [20] was applied to query sequences and identify improperly aligned pairs in the alignment, while Picard (http://broadinstitute.github.io/picard) was introduced to mark duplicate reads, which could represent sequencing depth and coverage. Copy number variations (CNVs) were identified not only around thyroid peroxidase gene (TPO) but genome wide using control-FREEC [21] and CREST [22] with default parameters, except for a window size of 5000, a step size of 2500, and the application of a control genome file of similar read depth. Identified CNVs were further inspected by Integrative Genomics Viewer (IGV) [23]. Chimeric reads which were mapped to multiple locations adjacent to possible breakpoint junctions were analyzed using Bowtie 2 v2.2.3 [24] so as to determine the exact positions of breakpoints and the form of genome rearrangements. The orientation of reading frame at each breakpoint was taken into consideration so as to predict possible form of gene fusion.

### PCR and sanger sequencing

To characterize the exact form of rearrangements and the position of breakpoints resolved from WGS, primer sets were developed (Additional file 1: Table S1) and the amplicons were devised to span the potential breakpoint junctions. A 50 μL PCR reaction mixture contained 20 ng human genomic DNA, 1× PCR buffer, 1.5 mM Mg^+^, 0.2 mM dNTP, 0.4 μM each primer (Sangon Biotech., China), and 1.5 U Taq polymerase. The singleplex PCR amplifications were performed in a GeneAmp PCR System 9700 (Applied Biosystems, USA) using the following conditions: initial incubation at 95 °C for 4 min; 32 cycles of denaturing at 94 °C for 10 s, 60 °C for 30 s, and 72 °C for 2 min with a final extension at 60 °C for 30 min. PCR products were subjected to a 1.5% agarose gel electrophoresis followed by purification and Sanger sequencing.

### Capillary electrophoresis (CE) validation

Primer sets suitable for CE were developed (Additional file 1: Table S1) and amplicons were devised to span the two breakpoint junctions, respectively. A single multiplex PCR system of 25 μL PCR reaction mixture containing 2 ng human genomic DNA, 1× PCR buffer, 1.5 mM Mg^+^, 0.2 mM dNTP, 0.4 μM each primer (forward primer labelled with FAM), and 1 U Taq polymerase was prepared. PCR amplification was performed under thermocycling conditions including initial incubation at 95 °C for 4 min; 30 cycles of denaturing at 94 °C for 10 s, 61 °C for 30 s, and 72 °C for 40 s with a final extension at 60 °C for 40 min. CE was performed according to our previous reports [25] and the fragments were separated and detected with ABI PRISM 3130xL Genetic Analyzer and analyzed with GeneMapper v3.2 software.

## Results

### TPOX with tri-allelic pattern in populations

**Fig. 1:**
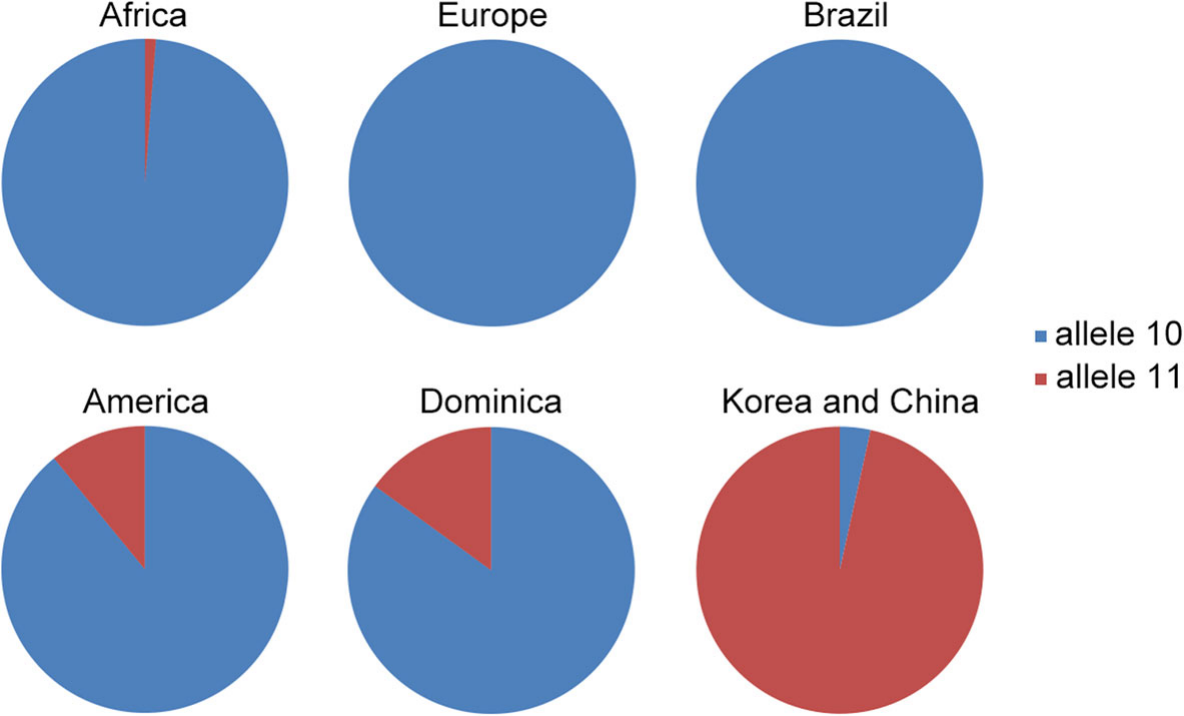
The number of extra alleles 10 and 11 at TPOX locus in unrelated individuals among populations. As for Europe, Belgium, France and Portugal are included

TPOX with tri-allelic pattern was investigated among populations on the basis of unrelated individuals from different geographic regions (Additional file 2: Table S2). The third allele was deemed either to be allele 10 or 11, while indefinite cases with the alleles 10 and 11 at TPOX locus were discarded. As shown in Fig. 1, nearly 98% (163 out of 165) of the extra allele of TPOX could be attributable to allele 10 in Africa. Nonetheless, a single allele of 11 still occurred in 2 samples with tri-allelic pattern at TPOX locus in African populations [5], which could denote a probability of slippage mutational event from the ancestral extra allele 10. In all unrelated samples from the Brazilian population and European populations including Belgium, France and Portugal, no definite extra allele of 11 has been observed. It is possible that cases with the extra allele 11 might be overlooked because the genotypes containing 10 and 11 were present at TPOX locus. The numbers of allele 11 appeared as the third allele rose in American and Dominican population while allele 10 still accounted for the major one. In this study, TPOX with tri-allelic pattern was observed in 6 unrelated individuals with a frequency of 0.059% in Chinese population, all of which contains an allele of 11 instead of allele 10. The same situation could be observed in other reported tri-allelic TPOX patterns in Chinese subjects (Additional file 2: Table S2). Taking unrelated Korean samples together into account, the vast majority of the third allele among Chinese and Korean populations was conversely observed to be allele 11.

### Investigation of the transmission of the third allele in family cases

In this study, genotypes of TPOX from family cases with true biological relationships in Chinese population were collected (Table 1) and the transmission of the extra allele was investigated. In case 2, the extra allele was observed to be not transmitted from the father to either of his daughters (Table 1). Meanwhile, previous reports indicated that males could transmit the third allele to their sons (case 6, 7 and 10 in Table 1) in Chinese population. The transmission of the extra allele at TPOX in these cases puts the assumption in dispute that the duplication of TPOX was on a chromosome X. As a matter of fact, Diaz has reported a transmission in Dominican population of the third allele 10 from the father to his son (case 19) [7], which is consistent with our observation in Chinese population. Based on these results, a presumption can be raised that the location of the third allele could not be on an X chromosome.

**Table 1:** STR genotyping of familial cases involving TPOX tri-allelic pattern in Chinese population

Case ^a^	Father	Son	Daughter	Mother	Reference
1	8	/	8,11,12	11,11,12 ^b^	This study
2	11,11,12 ^b^	/	10,11	10,11	This study
			11		
3	8,9	/	8,9	8,9,11	This study
4	8,11,12	8	/	/	This study
5	10,11,12	10,11	/	/	This study
6	9,11,12	8,11,12		8	[13]
7	/	8,9,11	/	8,9	[14]
8	8,11,12	8,11,12 ^c^		8	[16]
		8,11,12 ^c^			
9	8		8,9	9,11,12	[12]
10	8,8,11	8,9,11		9,11	[12]
11	8		8,11,12	8,11,12	[12]

^a^All subjects of tri-allelic patterns mentioned here are into the type 2 pattern

^b^The subject which undertakes next-generation sequencing and breakpoint analysis

^c^The gender of the child is not obtained in this study

### Determination of the location of the extra allele at TPOX

**Fig. 2:**
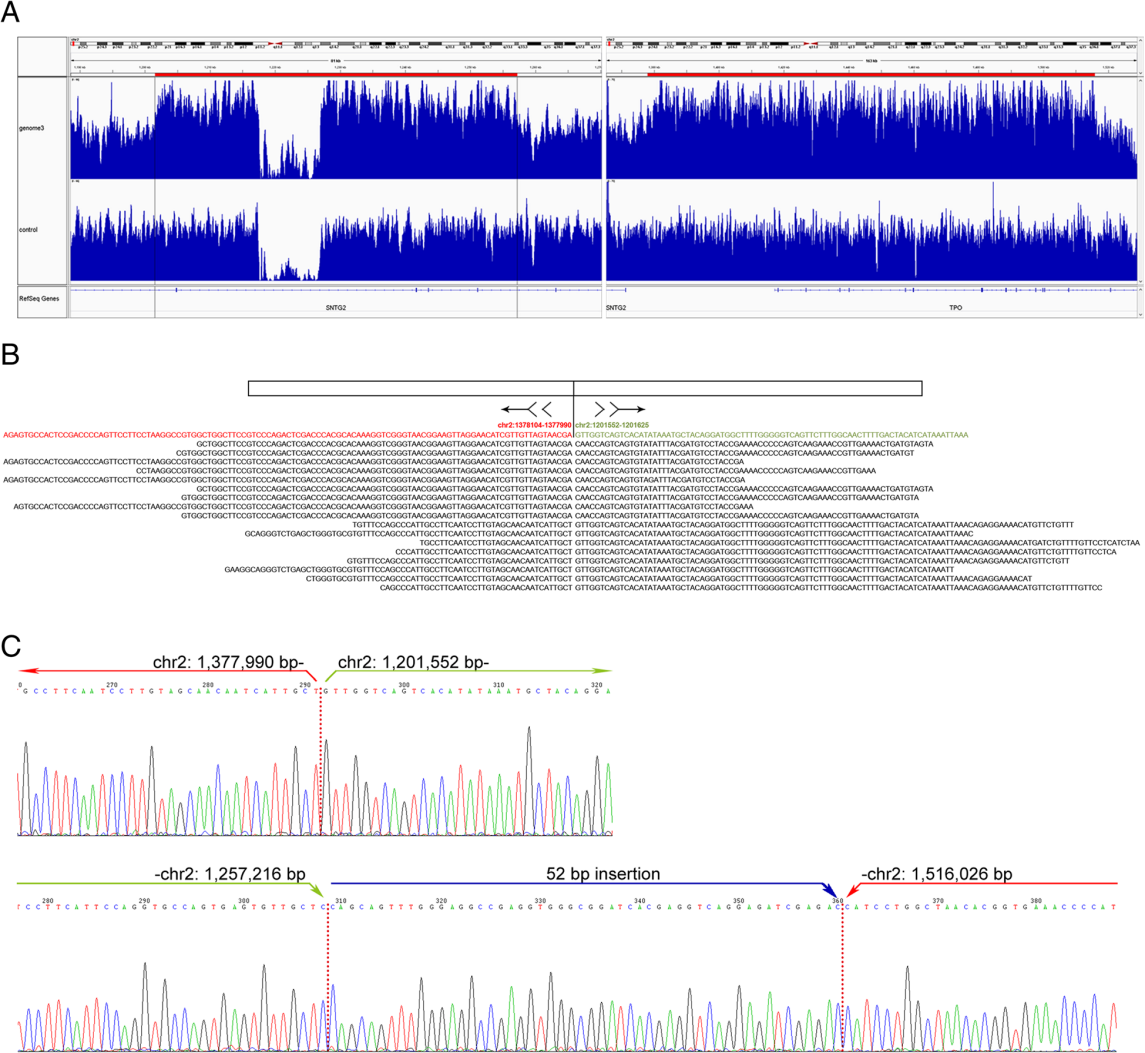
The determination of the genomic location of extra copy of TPOX. **a** The read depth of sample1 (genome 3) compared to that of a normal genome (control) using IGV. Left panel: the region (position chr2: 1,201,500-1,257,500 bp) with a duplication of SNTG2; Right panel: the region (position chr2: 1,377,500-1,509,999 bp) with a duplication of TPO gene. A pseudo deletion happens in SNTG2 in both genomes, which could be attributable to high G-C content therein. **b** A total of 18 split reads (of half of the read depth) was observed to be concurrently aligned to the fused region. The vertical solid line signposts the right breakpoint junction where the partial duplication of TPO joins in an inverted orientation to the region of SNTG2 as indicated by the arrows. **c** The breakpoints (red dashed lines) and flanking sequences. PCR products in Additional file 4: Fig. S2 were used for Sanger sequencing, respectively. The upper panel indicates the right breakpoint junction and its flanking regions. The lower panel indicates the left breakpoint junction where a 52 bp sequence was inserted in-between

To determine the rearrangement of TPO in human genome, WGS was performed on two samples, one from a male and another from a female. The analysis of structural variations revealed an extra copy covering a continuous region of ~ 138 Kb at TPO gene (Fig. 2a). Alignment of either soft-clipped or hard-clipped reads around 5′ end of this duplication revealed a definite fusion, in which the breakpoint at position chr2: 1,377,990 bp was inversely connected to the position chr2: 1,201,552 bp (Fig. 2b). In contrast, no chimeric reads from another end of this duplication were obtained in two samples. The analysis using 3 pairs of mate reads implied that another breakpoint junction of this duplication occurred approximately at chr2:1,515,800 bp and chr2:1,257,200 bp (Additional file 3: Figure S1). In fact, an extra copy in syntrophin gamma 2 gene (SNTG2) covering a region of ~ 56 Kb was observed (Fig. 2a), which can demonstrate that the breakpoint approximately at chr2:1,257,200 bp is located downstream at the position chr2: 1,201,552 bp.

To validate the breakpoints revealed by bioinformatics analysis, PCR was undertaken using two primer sets designed outside the two potential breakpoint junctions. Bright bands of identical size were obtained in two samples with tri-allelic pattern at TPOX (Additional file 4: Figure S2), confirming the same way of chromosomal rearrangements. By aligning results from sequencing with the reference genome, the exact form of breakpoint junctions was determined (Fig. 2c and d). The 5′ end of the intragenic copy in TPO (chr2: 1,377,990 bp) was inversely fused with the position chr2: 1,201,552 bp. Meanwhile, for another breakpoint, a sequence of 52 bp was inserted between the end of the duplicated region in SNTG2 (chr2: 1,257,216 bp) and the end of the duplication in TPO (chr2: 1,516,026 bp), which might interfere with the generation of reads with complete fusion sequences in WGS.

**Fig. 3:**
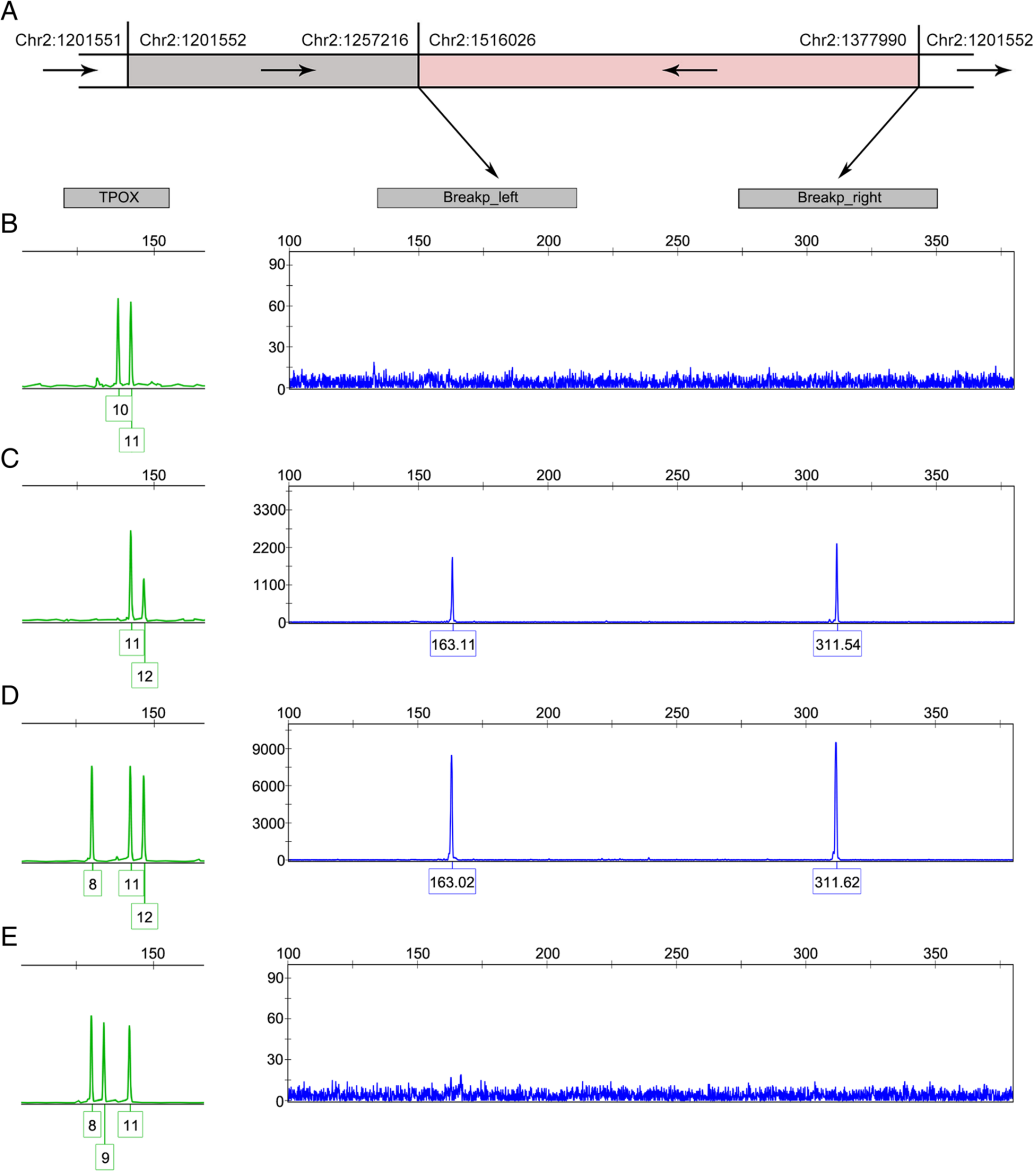
The detection of two breakpoints in unrelated individuals with tri-allelic pattern at TPOX. **a** A schematic diagram of the duplication and rearrangement of the extra copy of TPOX. The gray region represents a duplication of SNTG2 and the pink region represents a duplication of TPO. The arrows indicate the direction of genes on chromosome 2. **b-e** The detection of two breakpoint junctions by CE in a normal individual (**b**) and three unrelated individuals with tri-allelic pattern at TPOX (**c**, **d**, and **e**)

Consequently, the conjectural organization of the rearrangement events on chromosome 2 was deduced (Fig. 3a) taking sequence alignments and orientations into account comprehensively. The identical 52 bp insertion into the left breakpoint junction has also been validated among both samples, which processes a homologous sequence with Alu elements. As a matter of fact, such short insertions often take place between the two sides of the duplication, which plays a similar role to nonhomologous end-joining (NHEJ) [26] or microhomology-mediated break-induced replication (MMBIR) [27]. In this case, the 52 bp insertion could result in a prior disposure of a non-allelic homologous recombination (NAHR) [28] of the ~ 56 Kb intragenic tandem duplication in SNTG2 as the potential hotspot of recombination [29] for TPOX tri-allelic pattern.

### The location of the extra copy of TPOX in unrelated individuals

As the presentation of genomic rearrangement appeared to be homogeneous in 2 samples, it might well corroborate that the breakpoint junctions of such specific organization of genomic segments on chromosome 2 would be shared in the majority of three-banded samples. To investigate the universal nature of it in unrelated individuals, CE was applied to analyze PCR products amplified using two primer pairs spanning each breakpoint junction in both a normal sample and all other 4 samples with TPOX tri-allelic patterns in this study (Fig. 3b-e). In accordance with our previous conjecture, no breakpoint junction was detected in the normal sample. The possibility of a hidden sample of type 2 tri-allelic pattern with three identical alleles was ruled out for the daughter with the homozygous allele 11 in family case 2. Consistently, both breakpoints were detected at expected size in 3 out of 4 samples either with 2:1 (Fig. 3c) or 1:1:1 peak intensity (Fig. 3d). However, an exception was observed in one of the subject (Fig. 3e) in which neither of the breakpoints was detected. Generally, the consistent presence of such chromosomal rearrangements could be attributable to the identical origin from the ancient ancestral extra copy. Meanwhile, different forms of chromosomal rearrangement do exist among populations. For other TPOX tri-allelic cases in not only Chinese subjects but other populations, it is still required to use the primer sets developed in this study to verify the fusion of the third allele at TPOX locus in tri-allelic subjects.

## Discussion

In this study, we looked into tri-allelic cases at TPOX loci among different populations. As allele 10 is found overwhelming as the third allele in African and European regions, allele 11 seizes a dominant share in our study as well as in East Asia. In fact, the number of unrelated individuals with tri-allelic pattern at TPOX should be underestimated in the populations, since cases of indistinguishable third alleles have been excluded in this study. Additionally, three alleles with the same number of repeat units at TPOX locus can result in the omission of the tri-allelic pattern. As a result of random mutational events, an extra allele 9 or 12 might also be present at TPOX locus with tri-allelic pattern [1]. Such random mutational events would be quite rare among populations since the mutation rate of TPOX stays at very low level compared to other loci [30]. Since a large number of unrelated individuals among African populations appear to harbor the extra allele of TPOX, the proportion of individuals with tri-allelic pattern at TPOX in such population would stay flat or even increase if the fertility rate stays high. On the other side, for other populations whose frequency of type 2 TPOX tri-allelic patterns is rather low, it might be further attenuated from generation to generation given a low birth rate.

Events of chromosomal rearrangement and slippage mutation are critical in the generation of tri-allelic pattern. The existence of type 2 tri-allelic pattern is commonly attributable to a result of chromosomal rearrangement. While chromosomal aneuploidy could lead to an extra dose of amplification of the involved locus, a more restricted range of duplication could more frequently occur. Furthermore, a de novo third allele could be generated based on a slippage mutation of the ancestral allele. The extra allele 10 of TPOX is thought to be originated from Bantu groups in Africa and the allele 11 results from the slippage mutation of allele 10. Therefore, ancestors migrating from Africa to Asia should harbor the allele 11 since the allele 11 accounts for an overwhelming majority of the extra allele in East Asian populations. The extra allele 10 of TPOX in a Korean subject might be from the slippage mutation of allele 11. Likewise, the configuration of the tri-allelic pattern at TPOX in other populations summarized in this study might attribute to their population structures.

The transmission of the third allele between generations in the Chinese population was observed to conform to the genetic pattern of autosomal STR loci, holding an inconsistency to the previous hypothesis that the allele is located on chromosomal X. Certainly, the location of the extra allele of TPOX in other populations might be different from that in Chinese population. However, the allele 11 from the slippage mutation of allele 10 can reasonably demonstrate the common ancestry origin. Therefore, the determination of the location of the extra allele at TPOX locus can shed light on its transmission from parents to their offspring and help the statistical interpretation of STR genotyping in paternity testing.

WGS was performed on 2 unrelated individuals to reveal that the extra copy of tri-allelic pattern at TPOX was inserted into chromosome 2 instead of chromosome X, while the exact form of rearrangement in both subjects was conjectured. The homogeneous characteristics of the third allele of TPOX in these two samples could imply their identical origin of duplication and rearrangement. Also, as the inverted duplication of the extra allele has been inserted ~ 407 kb away from the authentic TPOX locus on chromosome 2, it is possible for the third allele to segregate independently from the authentic one [5]. This chromosomal rearrangement might not impair the physiological growth of individuals, since the tri-allelic pattern at TPOX locus can spread among populations. Nonetheless, it still remains bewildering that the tri-allelic pattern at TPOX has invariably been transmitted to daughters than sons in previous studies among different populations [5, 7, 9], which might suggest a potential impact of such chromosomal rearrangement on the activity of Y-sperms.

De novo rearrangement events would still take place, even though at an extensively low frequency, in bi-allelic individuals in populations to generate a tri-allelic pattern at TPOX locus. Interestingly, an exception has been observed by coincidence in this study that neither of the breakpoints was detected (Fig. 3e), raising the occurrence of the randomness during the rearrangement events concerning the third copy of TPOX in populations. Another random duplication generating the extra allele 8 or 10 at TPOX was detected with at least a 1.59 Mb surrounding duplicon in a previous study [31]. As TPOX allele 8 and 11 account for at least 70% allelic types among populations, the sporadic duplication and genomic rearrangement of TPO gene occurs more likely in individuals with the alleles 8 or 11. Although it appears possible that such rare rearrangement could also be transmitted from parents to their off-springs, the frequency could very likely be diluted and even eliminated from generation to generation given its small population proportion.

As for CNVs in genomic regions containing STR loci commonly used in forensic genetics, duplications were most frequently reported at chromosome 2 encompassing the TPOX locus. A total of 30 cases of copy gains encompassing TPOX were claimed at the Database of Genome Variants (DGV) website [32], with the size of duplication ranging from 6 to 860 Kb. Concerning genomes of samples with potential clinical indications, 11 duplications could be identified by clinical array CGH analysis at the International Standards for Cytogenomic Arrays (ISCA) database with pathological phenotypes including global development delay, cerebral palsy and so on [33]. Repnikova et al. also reported 14 cases of duplication at chromosomal 2p25.3 by array CGH [34]. Such duplication events could result in tri-allelic profiles, although most do not agree with the CNV we identified in this study. A similar duplication to our study has been detected in TPO in 2 variants as nssv543960 [35] and nssv1150389 [36], yet neither of them was reported along with the identical CNV in SNTG2. Although the existence of CNVs could often raise concern on potential diseases [37], type 2 tri-allelic patterns at TPOX, as noted, could be detected in phenotypically normal individuals, and no correlation has been established between the extra duplication with any predicted or known pathological phenotypes.

## Conclusion

In this study, we discussed the major characteristics of tri-allelic patterns at TPOX. Instead of the extra allele 10 in African populations, the main third allele at TPOX with tri-allelic pattern is allele 11 in Chinese and Korean populations. Whole genome sequencing was performed, revealing the universal form of genomic rearrangements on chromosome 2 of the extra copy, whose breakpoints were further validated in most samples. Meanwhile, the transmission of the third allele from parents to offspring is demonstrated. Therefore, the breakpoints of the ancestral extra copy are defined, which shows evidence of its inheritance from African populations. Since only a few samples were studied here, not a precise percentage of the third allele carrier at TPOX could be counted here, yet the simple validation method provided here could help identify rearrangements of the same origin in other labs. De novo chromosomal rearrangements at TPO gene randomly occurring in populations could also be recognized. Although the general frequency of TPOX tri-allelic patterns is low, it still calls for more efforts to discern tri-allelic patterns in allelic calling and to fully count the frequencies in different geographical regions, so that the allele transmission is clear from parents to children, and the statistical interpretation could be enhanced during forensic practice.

## Additional files

Additional file 1: **Figure S1**. Three mate reads were observed in NGS data. These 3 reads annotated using➀, ②, and ③ in the left panel were mated to 3 reads using the same annotations in the right panel, respectively. The reads in the left panel is located at the end of the duplication of SNTG2 and reads in the right panel is located at the end of the duplication of TPO (TIF 418 kb)

Additional file 2: **Figure S2**. The detection of two potential breakpoints by agarose gel electrophoresis. PCR amplification was performed on genomic DNA from a normal individual (Normal) and two unrelated individuals with tri-allelic pattern at TPOX undergoing WGS analysis (Sample1 and Sample2), respectively, using indicated primer set. PCR products were subjected to agarose gel electrophoresis. A DNA ladder was used as the marker (TIF 393 kb)

Additional file 3: **Table S1**. Primer sets for breakpoint sequencing and breakpoint validation in this study (DOCX 15 kb)

Additional file 4: **Table S2**. The number of the third allele of unrelated individuals with tri-allelic TPOX pattern (DOCX 26 kb)

## Abbreviations

BWA: Burrows-Wheeler Aligner
CE: Capillary electrophoresis
Chr: Chromosome
CNV: Copy number variation
CPI: Combined paternity index
IGV: Integrative Genomics Viewer
MMBIR: Microhomology-mediated break-induced replication
NAHR: Non-allelic homologous recombination
NGEJ: Nonhomologous end-joining
PCR: Polymerase chain reaction
SNTG2: Syntrophin gamma 2 gene
TPO: Thyroid peroxidase gene
WGS: Whole-genome sequencing
