# Supplementary figures and images for: Characterization of the extra copy of TPOX locus with tri-allelic pattern
Source: BMC Genet. 2019 Feb 14;20:18. doi: 10.1186/s12863-019-0723-2 (PMC6376737; doi:10.1186/s12863-019-0723-2)

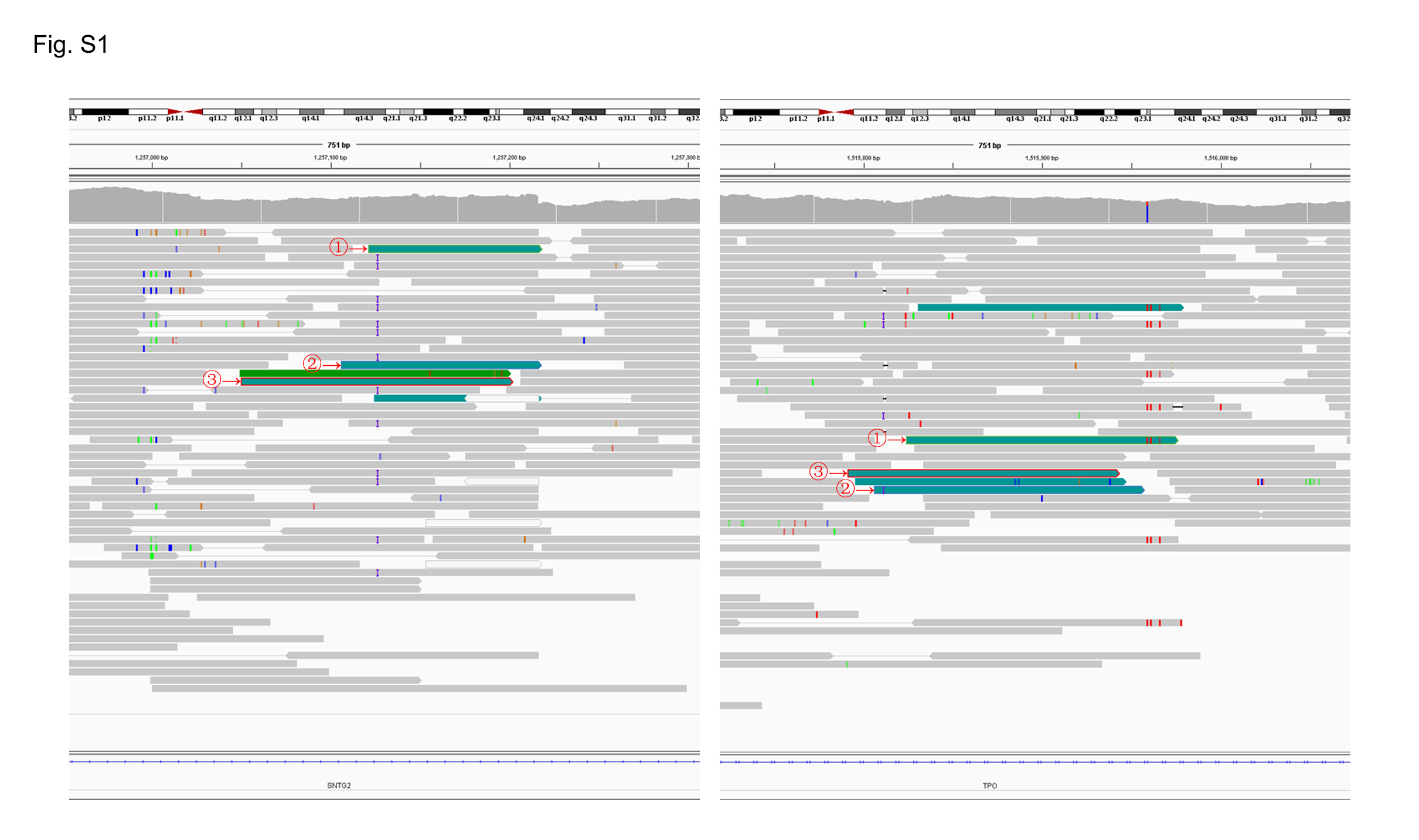

Supplement: Supplementary file 1 — Figure S1. Three mate reads were observed in NGS data. These 3 reads annotated using➀, ②, and ③ in the left panel were mated to 3 reads using the same annotations in the right panel, respectively. The reads in the left panel is located at the end of the duplication of SNTG2 and reads in the right panel is located at the end of the duplication of TPO (TIF 418 kb) [file 12863_2019_723_MOESM1_ESM.tif]

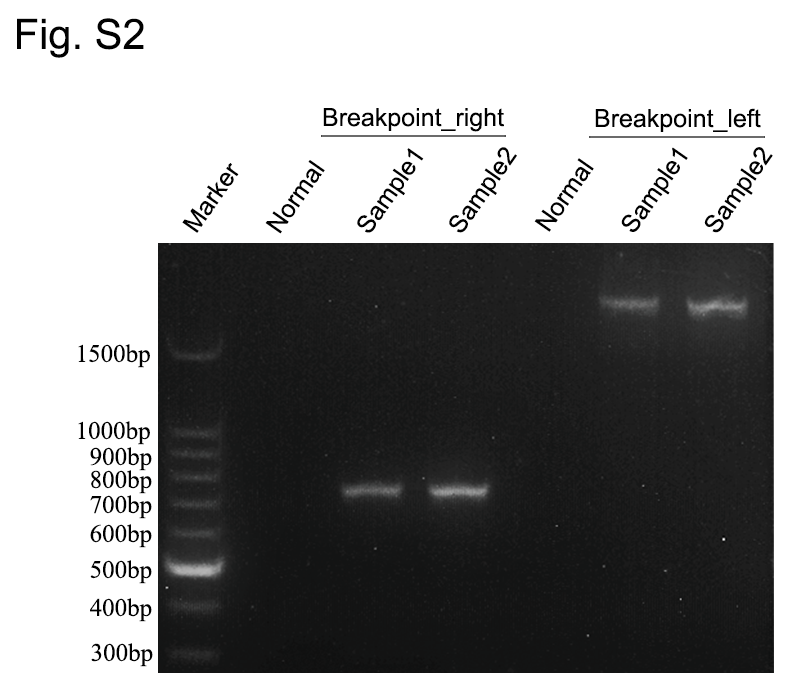

Supplement: Supplementary file 2 — Figure S2. The detection of two potential breakpoints by agarose gel electrophoresis. PCR amplification was performed on genomic DNA from a normal individual (Normal) and two unrelated individuals with tri-allelic pattern at TPOX undergoing WGS analysis (Sample1 and Sample2), respectively, using indicated primer set. PCR products were subjected to agarose gel electrophoresis. A DNA ladder was used as the marker (TIF 393 kb) [file 12863_2019_723_MOESM2_ESM.tif]
