# Supplementary material for: Characterization of the extra copy of TPOX locus with tri-allelic pattern
Source: BMC Genet. 2019 Feb 14;20:18. doi: 10.1186/s12863-019-0723-2 (PMC6376737; doi:10.1186/s12863-019-0723-2)
Supplement: Supplementary file 3 — Table S1. Primer sets for breakpoint sequencing and breakpoint validation in this study (DOCX 15 kb) [file 12863_2019_723_MOESM3_ESM.docx]

**Table S1** Primer sets for breakpoint sequencing and breakpoint validation in this study.

|  |  | Sequence (5'-3') | Product size (bp) |
| --- | --- | --- | --- |
| Sanger sequencing | Breakpoint_left-F | ACTCACAGAACGTCTCAATGCTG | 1862 |
|  | Breakpoint_left-R | TGTCTCCGTGGTGATTTACAGAC |  |
|  | Breakpoint_right-F | CACAGTGACCACGGTTTCTATGAC | 665 |
|  | Breakpoint_right-R | AGCAGAGCCTCGTGATTCTCATC |  |
| CE validation | Breakpoint_left-F | Fam-GAATGATTCACTATTGCCAACTCC | 163 |
|  | Breakpoint_left-R | GAGATGGGGTTTCACCGTGTTAG |  |
|  | Breakpoint_right-F | Fam-TGACGGAGCCCTTTCTGAGTG | 310 |
|  | Breakpoint_right-R | GGTTGCAAAAGCATCTGCATC |  |
