# Supplementary material for: Characterization of the extra copy of TPOX locus with tri-allelic pattern
Source: BMC Genet. 2019 Feb 14;20:18. doi: 10.1186/s12863-019-0723-2 (PMC6376737; doi:10.1186/s12863-019-0723-2)
Supplement: Supplementary file 4 — Table S2. The number of the third allele of unrelated individuals with tri-allelic TPOX pattern (DOCX 26 kb) [file 12863_2019_723_MOESM4_ESM.docx]

**Table S2** The number of the third allele of unrelated individuals with tri-allelic TPOX pattern

|  | Geographic region | Number of allele 10 | Number of allele 11 | References |
| --- | --- | --- | --- | --- |
|  | Africa | 163 | 2 | [1] |
|  | America | 49 | 6 | [2] ^a^ |
|  | Dominica | 17 | 3 | [3] |
|  | Europe ^b^ | 8 | 0 | [2] |
|  | Brazil | 69 | 0 | [2,4] |
|  | Korea | 1 | 9 | [2,5] |
|  | China | 0 | 20 | [2,6–12] and this study |

^a^ Concerning data obtained at STRbase website, only unrelated subjects at with a definite extra allele of 10 or 11 were included. Under circumstances that both allele 10 and 11 occurred in the genotype calling of an individual, the case was excluded in this study.

^b^ Belgium, France and Portugal were included.

**References**

[1] A.B. Lane, The nature of tri-allelic TPOX genotypes in African populations, Forensic Sci. Int. Genet. 2 (2008) 134–137. doi:10.1016/j.fsigen.2007.10.051.

[2] C.M. Ruitberg, D.J. Reeder, J.M. Butler, STRBase: a short tandem repeat DNA database for the human identity testing community., Nucleic Acids Res. 29 (2001) 320–322. doi:10.1093/nar/29.1.320.

[3] V. Díaz, P. Rivas, A. Carracedo, The presence of tri-allelic TPOX genotypes in Dominican Population, Forensic Sci. Int. Genet. Suppl. Ser. 2 (2009) 371–372. doi:10.1016/j.fsigss.2009.09.021.

[4] J.B. Picanço, P.E. Raimann, C.H.A.S. Da Motta, R. Rodenbusch, L. Gusmão, C.S. Alho, Identification of the third/extra allele for forensic application in cases with TPOX tri-allelic pattern, Forensic Sci. Int. Genet. 16 (2015) 88–93. doi:10.1016/j.fsigen.2014.11.016.

[5] S.Y. Yoo, N.S. Cho, M.J. Park, K.M. Seong, J.H. Hwang, S.B. Song, M.S. Han, W.T. Lee, K.W. Chung, A large population genetic study of 15 autosomal short tandem repeat loci for establishment of Korean DNA Profile Database, Mol. Cells. 32 (2011) 15–19. doi:10.1007/s10059-011-2288-4.

[6] Q. Liu, Y. Lai, D. Lv, H. Zhao, Y. Chen, W. Chen, Study of genetic polymorphism and tri-allelic pattern at TPOX, Evid. Sci. 18 (2010) 748–749. doi:10.3969/j.issn.1674-1226.2010.06.010. (in Chinese)

[7] H. Li, X. Li, F. Lan, L. Du, J. Lu, T. He, A. Yin, Analysis of genetic relationship in a tri-allelic case, Chinese J. Forensic Med. 28 (2018) 524–526. (in Chinese)

[8] Z. Liu, Y. Yang, J. Tong, X. Zhai, Triallelic STR Loci in Routine Forensic Analysis, Forensic Sci. Technol. 43 (2018) 148–151. doi:10.16467/j.1008-3650.2018.02.012. (in Chinese)

[9] Y. Zhang, H. Huang, Analysis on 3 tri-allelic variant cases of the STR TPOX locus, J. Nantong Univ. (Medical Sci. 33 (2013) 503–505. (in Chinese)

[10] F. Chen, J. Chen, Y. Fu, Q. Huang, H. Wen, 7 three-banded variant cases of the STR loci, Fa Yi Xue Za Zhi. 30 (2014) 215–216. doi:10.3969/j.issn.1004-5619.2014.02.20. (in Chinese)

[11] Y. Liu, H. Ren, Statistics analysis of tri-allelic patterns in STR loci, J. Forensic Med. 29 (2013) 444–446. (in Chinese)

[12] D. Zhou, X. Hu, T. Zhang, W. Wan, J. Yu, Z. Liu, L. Yu, A case of tri-allele in TPOX was detected using Goldeneye 20A Kit for the first time, China Med. Her. 15 (2018) 146–149. (in Chinese)
